# Host transcriptomic signatures of tuberculosis can predict immune reconstitution inflammatory syndrome in HIV patients

**DOI:** 10.1002/eji.202249815

**Published:** 2022-04-27

**Authors:** Stanley Kimbung Mbandi, Hannah Painter, Adam Penn‐Nicholson, Asma Toefy, Mzwandile Erasmus, Willem A. Hanekom, Thomas J. Scriba, Rachel P.J. Lai, Suzaan Marais, Helen A. Fletcher, Graeme Meintjes, Robert J. Wilkinson, Mark F. Cotton, Savita Pahwa, Mark J. Cameron, Elisa Nemes

**Affiliations:** ^1^ South African Tuberculosis Vaccine Initiative (SATVI) Institute of Infectious Disease and Molecular Medicine and Division of Immunology Department of Pathology University of Cape Town Cape Town South Africa; ^2^ Department of Infection Biology London School of Hygiene & Tropical Medicine London UK; ^3^ Department of Infectious Diseases Imperial College London London UK; ^4^ Wellcome Centre for Infectious Diseases Research in Africa University of Cape Town Observatory South Africa; ^5^ Institute of Infectious Disease and Molecular Medicine and Department of Medicine University of Cape Town Observatory South Africa; ^6^ Francis Crick Institute London UK; ^7^ Family Center for Research with Ubuntu Department of Pediatrics & Child Health Faculty of Medicine and Health Sciences Stellenbosch University Tygerberg South Africa; ^8^ Department of Microbiology and Immunology Miami Center for AIDS Research University of Miami Miller School of Medicine Miami Florida USA; ^9^ Department of Population & Quantitative Health Sciences Case Western Reserve University School of Medicine Cleveland Ohio USA

**Keywords:** Bacille‐Calmette‐Guerin, host biomarker, immune reconstitution inflammatory syndrome, transcriptomic signature, tuberculosis

## Abstract

Immune reconstitution inflammatory syndrome (IRIS) can be a complication of antiretroviral therapy (ART) in patients with advanced HIV, but its pathogenesis is uncertain. In tuberculosis (TB) endemic countries, IRIS is often associated with mycobacterial infections or Bacille‐Calmette‐Guerin (BCG) vaccination in children. With no predictive or confirmatory tests at present, IRIS remains a diagnosis of exclusion.

We tested whether RISK6 and Sweeney3, validated immune‐based blood transcriptomic signatures for TB, could predict or diagnose IRIS in HIV+ children and adults.

Transcripts were measured by RT‐qPCR in BCG‐vaccinated children and by microarray in HIV+ adults with TB including TB meningitis (TBM). Signature scores before ART initiation and up to IRIS diagnosis were compared between participants who did or did not develop IRIS.

In children, RISK6 and Sweeney3 discriminated IRIS cases from non‐IRIS controls before ART, and at diagnosis. In adults with TB, RISK6 discriminated IRIS cases from controls after half‐week on ART and at TB‐IRIS onset. In adults with TBM, only Sweeney3 discriminated IRIS cases from controls before ART, while both signatures distinguished cases from controls at TB‐IRIS onset.

Parsimonious whole blood transcriptomic signatures for TB showed potential to predict and diagnose IRIS in HIV+ children and adults.

## Graphical abstract

Host blood‐derived biomarkers based on tuberculosis parsimonious transcriptomic signatures showed potential to predict and diagnose immune reconstitution inflammatory syndrome upon initiation of antiretroviral therapy in children and adults with HIV.

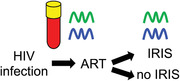

## Introduction

HIV‐associated immune reconstitution inflammatory syndrome (IRIS) is the paradoxical worsening or unmasking of an infection, neoplasm or inflammatory condition following initiation of antiretroviral therapy (ART). *Mycobacterium* spp. are most commonly associated with IRIS, however, IRIS can be caused by several other opportunistic viral, bacterial, and fungal infections including herpesviruses and *Cryptococcus neoformans* [1]. The incidence of IRIS is variable, with estimates between 5 and 50%, and, in most cases, IRIS develops within weeks to a few months following ART [2]. Bacille‐Calmette‐Guerin (BCG), the live‐attenuated vaccine against tuberculosis (TB), is frequently associated with IRIS in infants and young children [3]. In resource‐limited settings, the incidence of any IRIS in children initiating ART is 10–20% [3, 4, 5] but can be as high as 38% [6]. In 40 studies which cumulatively reported 1048 cases of paradoxical TB‐IRIS in adults, the pooled incidence of TB‐IRIS was 18% (range, 4–54) [7].

The clinical presentation of IRIS is highly heterogenous. Although two IRIS presentations have been recognized: “paradoxical” worsening of a known inflammatory condition or “unmasking” of a previously unrecognized infection, in most instances IRIS is a diagnosis of exclusion. No definitive diagnostic test for IRIS is available [1]. Whether the immunopathogenesis of IRIS is pathogen‐specific remains unknown, however, a key characteristic of IRIS is excessive, pathological inflammation following restoration of pathogen‐specific immunity during ART [1]. A 4‐week course of prednisone on initiating ART reduced TB‐IRIS incidence by 30% versus placebo in adults living with HIV (HIV+) [8]. Clinically applicable biomarkers for the diagnosis of IRIS or identification of individuals at risk of developing IRIS for closer monitoring and potentially better targeted prednisone prevention have yet to be found.

Since IRIS is commonly associated with *Mycobacterium tuberculosis* (M.tb) infection in adults and children, and *Mycobacterium bovis*‐derived BCG vaccination in children, we tested whether blood gene‐expression signatures developed to predict and diagnose TB could be applied to IRIS. We selected a TB risk (RISK6) and a TB diagnostic (Sweeney3) transcriptomic signature because they are concise (six or less transcripts) and have been widely validated in diverse cohorts. RISK6 is a six‐gene (GBP2, FCGR1B, SERPING1, TUBGCP6, TRMT2A, and SDR39U1) whole blood transcriptomic signature that predicts incident TB in M.tb‐infected individuals up to 12 months prior to TB diagnosis and demonstrated promising diagnostic performance for active TB [9, 10]. Sweeney3 is a three‐gene (GBP5, DUSP3, and KLF2) transcriptomic signature developed to diagnose active TB disease, which also performs well at predicting incident TB on prospectively collected samples [11, 12]. These signatures correlate with severity of lung pathology and potentially indicate treatment response [10, 12]. More recently, Sweeney3 was evaluated as a nonsputum triage diagnostic test in HIV+ people with a prototype cartridge assay (Xpert MTB Host Response, Cepheid) [13].

In this study, RISK6 and Sweeney3 were applied to a HIV+ paediatric IRIS cohort. Signature scores were determined in whole blood obtained at ART initiation and IRIS diagnosis to evaluate their ability to discriminate between IRIS cases and controls. The performance of both signatures was also re‐evaluated in silico in two published studies including adults on ART with TB‐IRIS [14] and TB meningitis IRIS (TBM‐IRIS) [15].

## Results and Discussion

**Figure 1:**
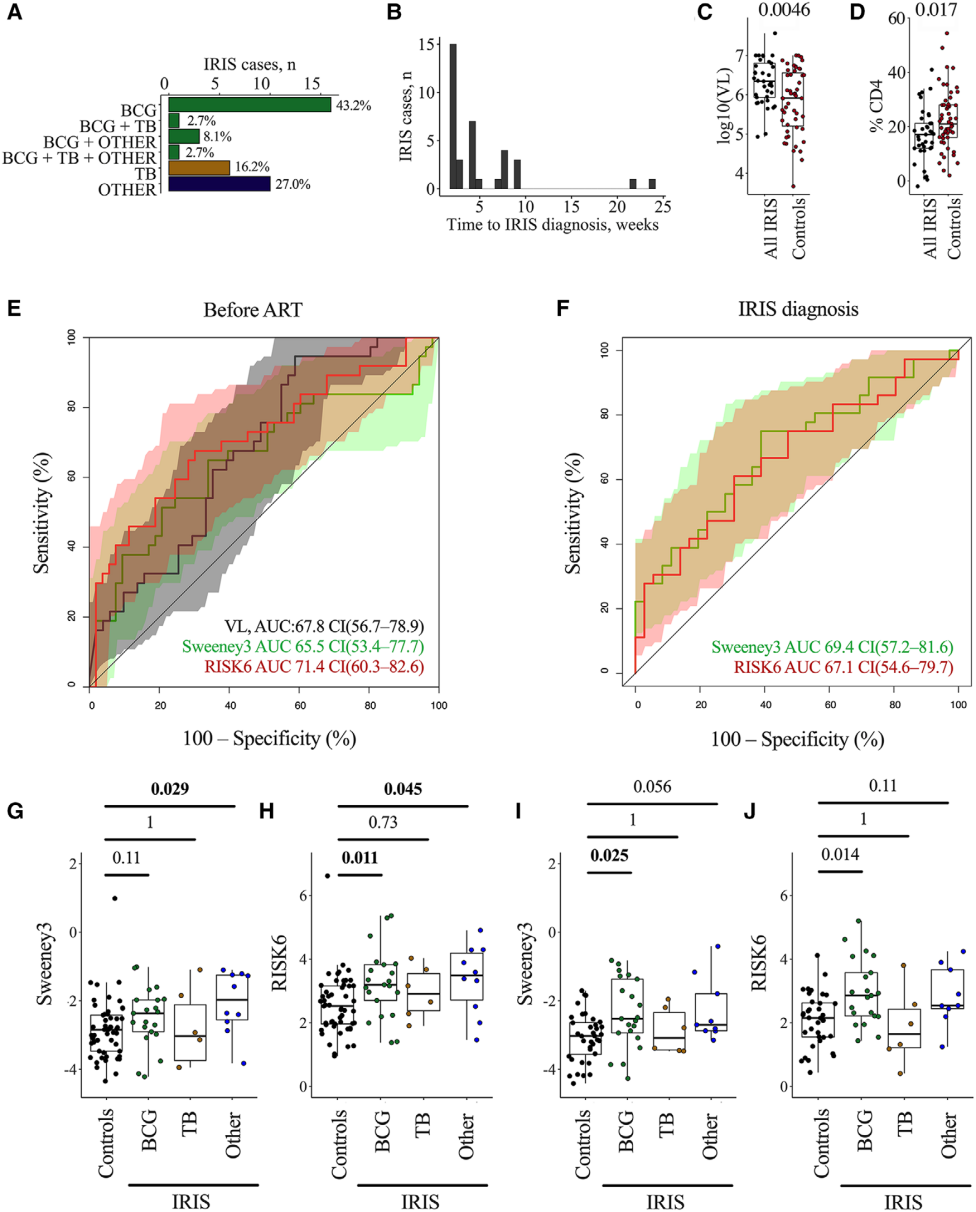
**Evaluation of RISK6 and Sweeney3 performance for discrimination between IRIS cases and controls in an HIV‐positive paediatric cohort**. Transcript signatures were quantified in whole blood by RT‐qPCR in HIV+ children at ART initiation and IRIS diagnosis. Distribution of (A) IRIS cases (total n = 37) by type and relative proportions, (B) time to IRIS diagnosis in weeks, (C) log10(VL), and (D) %CD4 at ART initiation in cases (n = 37) and controls (n = 53). (E) ROC curves depicting the performance of HIV viral load (VL, grey), Sweeney3 score (green), and RISK6 score (red) to discriminate between IRIS cases (n = 37) and controls (n = 53) at ART initiation. (F) ROC curves depicting performance of Sweeney3 (green) and RISK6 (red) to discriminate between IRIS cases (n = 36) and controls (n = 36) at IRIS diagnosis. Comparison of (G) Sweeney3 and (H) RISK6 scores in the subgroups of IRIS (BCG n = 21, TB n = 6, other n = 10) and all controls (n = 53) at ART initiation. Comparison of (I) Sweeney3 and (J) RISK6 scores in all controls (n = 36) and the subgroups of IRIS (BCG n = 21, TB n = 6, other n = 9) at IRIS diagnosis. “Other” cases of IRIS were oral candida, eczema, CMV colitis, molluscum contagiosum, cryptococcal meningitis, papular pruritic eruption, seborrheic dermatitis, tinea capitis, and zoster. Shaded areas in the ROC curves depict the 95% CI. The horizontal lines in the boxplots depict the median; boxes represent the IQR and whiskers the range; dots represent individual sample scores. *p*‐values were calculated using the Wilcoxon rank‐sum test with Bonferroni correction for multiple comparisons. Adjusted *p*‐values <0.05 were considered significant (bold text).

We evaluated the performance of two concise blood transcriptomic TB signatures to predict IRIS prior to ART initiation and to diagnose IRIS on presentation in a cohort of HIV+ infants and young children initiating ART. Among 198 participants in the larger cohort, 38 children (18.8%) developed IRIS. Of the 90 children included in this study, median age was 9.4 months (range, 2.0–65.5 months) and 51.1% were females. The attributed causes of IRIS [3] are shown in Fig. 1A. “Other” cases of IRIS were oral candida, eczema, CMV colitis, molluscum contagiosum, cryptococcal meningitis, papular pruritic eruption, seborrheic dermatitis, tinea capitis, and zoster. Median time to first IRIS event was 3.5 weeks (range, 2–24 weeks) after ART initiation (Fig. 1B). For participants who experienced multiple IRIS episodes, only data from the first IRIS event were included in this study. Despite the broad matching for these variables, children experiencing IRIS had higher HIV viral load (VL) (Fig. 1C) and lower %CD4 at ART initiation (Fig. 1D) than controls, as previously reported [3].

Both signatures significantly predicted IRIS cases and non‐IRIS controls on samples collected prior to ART initiation (Fig. 1E; RISK6 area under curve [AUC]: 71.4, 95% confidence interval (CI): 60.3–82.6, *p* = 0.00057; Sweeney3 AUC: 65.5, 95% CI: 53.4–77.7, *p* = 0.013) and at diagnosis (Fig. 1F; RISK6 AUC: 67.1, 95% CI: 54.6–79.7, *p* = 0.007; Sweeney3 AUC: 69.4, 95% CI: 57.2–81.6, *p* = 0.018). The performance of the two transcriptomic signatures was not significantly different from each other (roc.test, *p*‐value = 0.19).

We previously showed that increased HIV plasma VL is a significant baseline predictor of paediatric IRIS [3]. We determined whether combining VL and RISK6 signatures scores led to better discrimination than using the signature scores alone. We found no statistically significant incremental gain in biosignature performance when signature scores at baseline were combined with VL in multivariate regression analysis, AUC: 75.5, 95% CI: 64.9–86.1, roc.test *p* = 0.32 (Fig. 1E) [16].

Upon stratification by “type” of IRIS, both Sweeney3 and RISK6 signature scores were significantly higher in “other” IRIS cases than controls before ART, while only RISK6 was different in BCG‐IRIS compared with controls, after adjustment for multiple comparisons (Fig. 1G and H). At IRIS diagnosis, both signature scores were significantly different only between BCG‐IRIS and controls (Fig. 1I and J). Signature scores were not different at either of the time points between TB‐IRIS cases and controls, although with only six TB IRIS cases this comparison was based on a very small sample size.

Both RISK6 and Sweeney3 signatures contain IFN‐stimulated genes, which are upregulated in TB [17, 18] and in HIV; and correlate with inflammation [9, 12, 19]. Signature scores in the paediatric IRIS dataset likely reflect inflammation regardless of the underlying coinfection.

We also evaluated the performance of RISK6 and Sweeney3 in two published ART‐naïve HIV+ adult IRIS datasets on samples collected before ART initiation, on intermediate time points prior to and at diagnosis of IRIS [14, 15].

**Figure 2:**
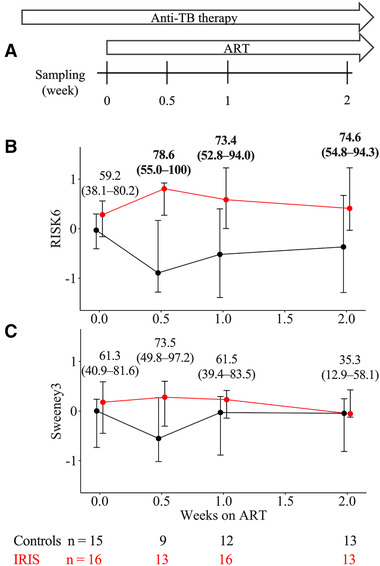
**Distribution of TB signature scores in TB‐IRIS patients and non‐IRIS controls**. Gene expression was quantified by microarray in whole blood from HIV+ adults with TB. Sweeney3 and RISK6 scores were computed on the normalised log2‐transformed transcript level intensities in samples collected at 0, half‐, 1‐, and 2‐weeks (median time to IRIS onset) on ART. (A) Schematic of the sampling timeline. Comparison of (B) RISK6 and (C) Sweeney3 scores in TB‐IRIS patients (n = 16) and non‐IRIS controls (n = 15). The AUC is depicted for each timepoint and is considered significant where the 95% confidence interval > 50 (bold text). The whiskers above and below represent the interquartile range of values and the dot indicates the median.

In the TB‐IRIS cohort (Fig. 2A), RISK6 scores were different between cases and controls at half‐ (AUC: 78.6, 95% CI: 55.0–100.0, *p* = 0.025), one‐ (AUC: 73.4, 95% CI: 52.8–94.0, *p* = 0.037), and 2‐weeks (median TB‐IRIS onset; AUC: 74.6, 95% CI: 54.8–94.3, *p* = 0.034) on ART, but not at ART initiation (Fig. 2B). Sweeney3 scores were not significantly different between cases and controls in this cohort at any time point (Fig. 2C).

**Figure 3:**
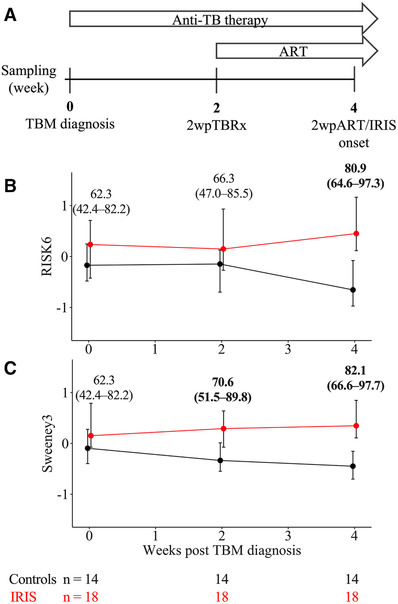
**Distribution of TB signature scores in patients with TBM with and without IRIS**. Transcript levels were quantified by microarray in whole blood from HIV+ adults with TBM. Sweeney3 and RISK6 scores were computed on the normalised log2‐transformed transcript level intensities in samples collected at TBM diagnosis, two weeks after tuberculosis treatment (2wpTBRx) when ART started, and two weeks after ART initiation (2wpART, typical time of IRIS onset). (A) Schematic of the sampling timeline. Comparison of (B) RISK6 and (C) Sweeney3 scores between controls (n = 14) and TB‐IRIS cases (n = 18). The AUC is depicted for each timepoint and is considered significant where the 95% confidence interval > 50 (bold text). The whiskers above and below represent the interquartile range of values and the dot indicates the median.

In the TBM‐IRIS cohort (Fig. 3A), neither signature discriminated between IRIS cases and controls at TBM diagnosis (Fig. 3B and C). At ART initiation, only Sweeney3 scores were different between cases and controls (AUC: 70.6, 95% CI: 51.5–89.8, *p* = 0.049, Fig. 3C). Both Sweeney3 and RISK6 scores were significantly higher in IRIS cases than controls at diagnosis (median onset 2 weeks after ART initiation; AUC: 82.1, 95% CI: 66.6–97.7, *p* = 0.0015 and AUC: 80.9, 95% CI: 64.6–97.3, *p* = 0.0024, respectively, Fig. 3B and C).

Systemic inflammation is a hallmark of IRIS and host gene expression profiling has been applied to characterise the underlying inflammatory processes [14]. However, no IRIS prognostic or diagnostic biomarker based on parsimonious transcriptional signatures has been described to date [1].

Unlike Sweeney3, the RISK6 TB signature was first identified in HIV‐uninfected, M.tb‐infected adolescents or adults, although its diagnostic performance has now been demonstrated in HIV+ adults [9, 20]. Here, we show that RISK6 could predict and diagnose IRIS in children, and could distinguish between adult cases and controls at diagnosis of both TB and TBM‐IRIS. Sweeney3 performance was similar to RISK6, except that it failed to distinguish between adults with TB‐IRIS and controls. These findings are remarkable considering the limitations of this study, which include small sample sizes of the three datasets; considerable heterogeneity in IRIS aetiology and presentation; and differences in age, which are associated with variation in immune maturity [21]. The modest but significant prognostic and diagnostic performance is also noteworthy considering that signature scores are affected by both HIV and TB disease [19] and that all HIV+ adults, including controls, also had TB disease.

Host genetics, antigen load, CD4 T‐cell count, and HIV VL independently or in concert, contribute towards IRIS [1]. It remains unproven whether a single factor, be it of pathogen or host origin, is solely implicated, but underlying each case of IRIS is heightened inflammation and hypercytokinemia involving both innate and adaptive immune responses. Most IRIS cases implicate mycobacteria and evidence from systems immunological analysis of whole blood transcriptomes reveal common immune pathways in the pathogenesis of TB and IRIS, including IFN signalling, inflammasome, and complement activation [14, 15, 22], and one or more genes in these pathways are often a component of the ever‐increasing list of biosignatures for TB [23]. RISK6 and Sweeney3 both include IFN‐stimulated genes and higher signature scores are in keeping with persistent inflammation observed in TB.  Changes in IFN‐stimulated genes during progressive Mtb infection, also involving dysregulation in the compliment system, have been well described [24, 25]. Our results suggest that these two TB signatures measure common inflammatory processes that are independent of the pathogen and immune maturation of the host, and may underlie similar immunopathological pathways in TB and IRIS.

Further studies are needed to compare the performance of RISK6 and Sweeney3 with that of other nonspecific markers of inflammation such as C‐reactive protein.

## Conclusions

Together, these findings show that whole blood transcriptomic biomarkers for TB could aid in the prognosis and diagnosis of IRIS in paediatric and adult HIV+ patients starting ART. Larger, prospective studies are required to assess predictive and diagnostic performance more accurately and to determine the clinical relevance of these findings. Development of point‐of‐care tests for TB which incorporate Sweeney3 and RISK6 is already underway and could benefit the management of IRIS if larger studies demonstrate clinical utility.

## Materials and methods

### Paediatric IRIS cohort

We nested this case‐control study in a larger previously described IRIS cohort from P1073, a study of the International Maternal, Pediatric and Adolescent Clinical Trial (IMPAACT) network [3]. The study was approved by the ethics committees of all seven clinical research sites, located in sub‐Saharan Africa and India, was conducted according to the Declaration of Helsinki and a parent or legal guardian of each participant provided written, informed consent.

ART‐naïve HIV+ children <72 months of age were recruited at ART initiation into an observational multicentred prospective clinical study between December 2010 and September 2013 and followed‐up for 48 weeks. Proof of BCG vaccination was required for infants up to 1 year of age. However, all children were recruited in TB endemic countries where universal BCG vaccination was recommended. The cohort for the current study comprised 90 ART‐naïve HIV+ infants and young children including 37 IRIS (43 episodes) and 53 non‐IRIS (control) individuals. Twenty‐one cases who experienced BCG‐IRIS, alone or in combination with TB or other pathogens, were matched to 21 controls by %CD4 at baseline (> or <25%), enrolment site, age (±6 months) and ART duration at IRIS diagnosis (±2 weeks). An additional 16 cases, who experienced IRIS associated with a variety of pathogens, were matched to 32 controls by enrolment site, age (±12 months), and ART duration at IRIS diagnosis (±4 weeks), based on availability of controls. Controls were pooled for all analyses reported here. IRIS was diagnosed according to the criteria proposed by Haddow et al. [26], as previously reported in details [3]. Briefly, IRIS was defined as an atypical or exaggerated infectious or inflammatory adverse event in the first 26 weeks after starting ART, which was not associated with treatment failure, adverse drug reactions, a newly acquired infection, noncompliance to ART or TB treatment. TB‐IRIS was diagnosed based on published case definitions [27]. As commonly observed in children, only two cases had microbiological confirmation of TB (details in [3]). Paradoxical and unmasking IRIS cases were pooled for all analyses reported here.

### RNA extraction and RT‐qPCR

Venous blood was collected prior to ART initiation from all children, during follow‐up (controls) and at IRIS diagnosis (cases) and stored in RNALater (Qiagen, Germany) at –80°C until use. RNA was extracted with the RiboPure‐Blood kit (Ambion, USA) and RT‐qPCR was performed to quantify RISK6 and Sweeney3 signature transcripts as previously described [9].

### Adult IRIS gene expression dataset

Publicly available whole blood transcriptomic datasets (Illumina human HT‐12 v4 BeadChip arrays) were obtained from the Gene Expression Omnibus (GEO) repository. The metadata, including the IRIS diagnosis, CD4 and HIV VL were available from the original study databases. The designs for both studies have been described previously [14, 15].

The TB‐IRIS dataset (GSE58411) [14] comprised hospitalised adults (>18 years old) with HIV‐associated TB (all forms). Patients were ART naïve at recruitment and were followed‐up longitudinally for development of TB‐IRIS. Tempus blood samples were obtained from 17 TB‐IRIS and 15 non‐IRIS patients at four time points: ART initiation, half‐, 1‐, and 2‐weeks after ART initiation (median time to IRIS onset).

The TBM‐IRIS dataset (GSE83892) [15] comprised hospitalised adults (>18 years old) with HIV‐associated TBM. Patients were ART naïve at recruitment and were followed‐up longitudinally for development of IRIS. PAXgene blood samples were obtained from 18 patients with IRIS (of whom 15 had TMB‐IRIS, 1 had pulmonary TB‐IRIS, and 2 had node TB‐IRIS) and 14 patients with TBM but no IRIS (controls) at three time points: TBM diagnosis, ART initiation, and at IRIS presentation or at 2 weeks after ART initiation in controls.

In the adult cohorts, IRIS was diagnosed based on published case definitions [27]. Briefly, paradoxical IRIS was defined as new or worsening clinical manifestations within 3 months of ART initiation in patients with previously diagnosed TB or TBM, who showed initial response to TB treatment and in whom alternative explanations for clinical deterioration were excluded.

### Statistical analysis

Signature scores for microarray and qRT‐PCR were obtained from normalized log_2_‐transformed MFI and *Ct* values, respectively. The microarray datasets from GEO were processed in R (R Core Team, 2019) and analysed using the MetaIntegrator package [28], which implements quantile normalization and provides a reproducible pipeline for pre‐processing microarray, eliminates the technical hurdles involved with gene annotation between different array generations and summarizes multiple probes MFI to gene‐level intensities. This same approach was implemented in the discovery of Sweeney3 [11] and when applying RISK6 to microarray [9]. The RISK6 and Sweeney3 signature scores were computed for RT‐qPCR as reported previously [9]. The AUC and receiver operating characteristic (ROC) curve were obtained using the Delong method with the pROC package [29]; 95% CIs were computed using 100 stratified bootstrap replicates. Multivariate logistic regression of IRIS status was modelled on RISK6 and VL using the *g* *lm* function in R and predicted probabilities were used to assess model performance with ROC curve. Statistical significance was tested by Wilcoxon rank‐sum test followed by Bonferroni correction for multiple comparisons and *p*‐values <0.05 were considered significant.

## Conflict of interest

TJS, APN, and WAH are coinventors of a patent of the RISK6 signature. The other authors declare no conflict of interest.

## Authors’ contributions

WAH, SM, GM, RJW, MFC, SP, MJC, and EN designed the studies and collected critical clinical information and specimens; APN, AT, and ME generated the data; SKM and HP analysed the data; SKM, HP, TJS, RPJL, HAF, GM, RJW, SP, and EN interpreted the results; SKM, HP, and EN wrote the manuscript, which was reviewed and approved by all authors.

### Peer review

The peer review history for this article is available at https://publons.com/publon/10.1002/eji.202249815

**Abbreviations**
AUC: area under curve
ART: antiretroviral therapy
BCG: Bacille‐Calmette‐Guerin
CI: confidence intervals
GEO: Gene Expression Omnibus
IMPAACT: International Maternal, Pediatric and Adolescent Clinical Trial
IRIS: immune reconstitution inflammatory syndrome
M.tb: *Mycobacterium tuberculosis*
ROC: receiver operating characteristic
TB: tuberculosis
TBM‐IRIS: TB meningitis IRIS
VL: viral load

## Data Availability

New data were generated and analysed in this study, which can be found in the following online repository,  https://doi.org/10.25375/uct.13602836.v1. Publicly available datasets on GEO (https://www.ncbi.nlm.nih.gov/geo/) were also analysed in this study (GSE58411 and GSE83892). Code and formular used to calculate signature scores are included in the original publications [9, 11].
